# The Role of Catastrophizing in Enhancing Pain Interference and Depressive Symptoms in Endometriosis: A Longitudinal Examination

**DOI:** 10.1002/ejp.70198

**Published:** 2025-12-26

**Authors:** Marta Spinoni, Cristian Di Gesto, Maria Grazia Porpora, Caterina Grano

**Affiliations:** ^1^ Department of Psychology Sapienza University Rome Italy; ^2^ Department of Maternal and Child Health and Urological Sciences Sapienza University of Rome Rome Italy

**Keywords:** chronic pelvic pain, depression, endometriosis, fear‐avoidance model, pain catastrophizing, pain interference

## Abstract

**Background:**

Endometriosis is a prevalent condition characterised by chronic pelvic pain, significantly impacting women's quality of life and well‐being. Pain catastrophizing is a cognitive tendency of exaggerated worrying, a sense of helplessness, and amplification of distressing thoughts in response to pain. According to the Fear‐Avoidance Model, catastrophizing contributes to prolonged pain interference and mood disorders. This longitudinal study examines the relationship between pain catastrophizing, pain interference, and depressive symptoms in patients with endometriosis.

**Methods:**

A sample of 128 cisgender women with endometriosis recruited from online patient associations completed self‐report measures assessing pain catastrophizing, pain interference, and depressive symptoms at baseline (T1) and after 6 months (T2). A mediation model examining the indirect effect of pain catastrophizing on depressive symptoms through pain interference was tested. The pelvic pain level was considered as a covariate in the model.

**Results:**

More than half of the sample reported depressive symptoms above the cut‐off. Pain catastrophizing at T1 significantly predicted depressive symptoms at T2. Pain interference partially mediated this relationship.

**Conclusions:**

Our findings indicate that heightened levels of catastrophizing correlate with increased pain interference, which, in turn, predicts elevated depressive symptoms over time. Findings suggest that by targeting catastrophizing tendencies, clinicians may help mitigate depressive symptomatology.

**Significance Statement:**

This longitudinal study investigates for the first time the impact of pain catastrophizing on pain interference and depressive symptoms over time in cisgender women with endometriosis. Findings indicated both a direct influence of pain catastrophizing on depressive symptomatology and an indirect effect via increased pain interference, in line with the Fear‐Avoidance Model. These findings underscore the importance of targeting pain catastrophizing tendencies in interventions aimed at improving the well‐being of patients affected by endometriosis.

## 1 Introduction

Endometriosis is a highly prevalent chronic disease that affects approximately 6%–10% of females of reproductive age (Reid et al. 2019; Singh et al. 2020). However, these percentages likely underestimate the true prevalence of the disease. In fact, endometriosis symptoms are frequently overlooked and normalised by primary care physicians and family members, resulting in delayed diagnosis and management (Sachedina and Todd 2020).

The most common and debilitating symptom of endometriosis is chronic pelvic pain, including dysmenorrhea (pain at menstruation), dyspareunia (pain at intercourse), and acyclic pain (Kotowska et al. 2021; Hsu et al. 2010; Moradi et al. 2014). Endometriosis significantly affects women's quality of life, interfering with multiple functional areas, including physical (Silva et al. 2024), sexual (Rossi et al. 2022; Pope et al. 2015), and relational (Culley et al. 2013) domains. Job productivity is also negatively impacted by an increased number of sick days and pain‐related work disturbances (Soliman et al. 2017; Hansen et al. 2022). Remarkably, this condition also has a detrimental impact on mental health and well‐being (Wang et al. 2021; Moore et al. 2023; Laganà et al. 2017), with depressive symptoms affecting up to 65% of women with endometriosis (Gambadauro et al. 2019). Moreover, as highlighted in recent systematic reviews (van Barneveld et al. 2022; Gambadauro et al. 2019; Lorençatto et al. 2006), patients with endometriosis, especially those with chronic pelvic pain, experience significantly higher levels of depression when compared to healthy controls.

The psychological vulnerability to depressive symptoms may be further complicated by pain catastrophizing (Trudel and Cormier 2024), a cognitive tendency characterised by exaggerated worrying, a sense of helplessness, and amplification of distressing thoughts in response to pain (Sullivan et al. 2001), often reported among patients with chronic pain (Petrini and Arendt‐Nielsen 2020). According to the Fear‐Avoidance Model of chronic pain (Vlaeyen and Linton 2000), individuals who use catastrophizing as a negative coping mechanism to deal with pain are at increased risk for emotional and behavioural dysfunctional responses, which, in turn, result in prolonged pain interference and mood disorders (Sisco‐Taylor et al. 2022). Indeed, a large body of evidence has demonstrated the crucial role of pain catastrophizing in maintaining pain, exacerbating functional disability, and further increasing psychological distress in various chronic pain populations (Slepian et al. 2020; Edwards et al. 2016; Ben Tekaya et al. 2024; Quartana et al. 2009). For instance, in patients with low back pain, pain catastrophizing was associated with altered pain sensitivity (Meints et al. 2019) and reduced physical functioning (Pierobon et al. 2020). In chronic pain patients, regardless of pain location, it has been positively linked to higher pain intensity, increased affective distress, and more severe depressive symptoms (Roth et al. 2005). Similarly, in patients with neuropathic pain, pain‐related helplessness has been associated with greater pain intensity and disability (Sullivan et al. 2005). In the context of women with endometriosis, catastrophizing has been identified as a predictor of pain intensity at 1‐year follow‐up (Martin et al. 2011) and, in cross‐sectional research, as a moderator of the relationship between pain severity and depressive symptoms (Zarbo et al. 2024).

Despite these findings, a comprehensive examination of the longitudinal relationship between catastrophizing, pain interference, and depressive symptoms in patients with endometriosis is lacking.

In this study, an unprecedented attempt was made to investigate whether pain catastrophizing would be associated with depressive symptoms via pain interference. Specifically, pain catastrophizing was expected to directly correlate with pain interference, such that more frequent catastrophizing would be associated with more pain interference. In turn, pain interference would longitudinally predict higher levels of depressive symptoms 6 months later. Moreover, the direct impact of catastrophizing on depressive symptoms over time was also hypothesised.

This research aimed to provide a deeper understanding of the psychological aspects of the endometriosis experience, with the goal of informing potential interventions to enhance the well‐being of patients affected by this condition.

## 2 Method

### 2.1 Participants and Procedures

The current study utilises data from a multidisciplinary investigation on pelvic pain in patients with endometriosis, conducted between February 2023 and July 2023. The original sample comprised 301 participants recruited at the Endometriosis and Chronic Pelvic Pain Outpatient Service of Policlinico Umberto I University Hospital of Rome and through social media platforms (e.g., online patient associations and Facebook thematic groups on endometriosis). A preliminary cross‐sectional study utilising the entire sample examined body‐related factors in the pathway from pain severity to depression, employing a cross‐sectional design (Spinoni et al. 2024).

In this second study, a total of 201 cisgender women participated in the initial assessment. Participants were recruited from two sources: (a) a subsample of 160 women with endometriosis who had participated in a previous study conducted in an outpatient gynaecology clinic and through patient advocacy groups, and (b) an additional 41 women recruited through online announcements and social media, yielding a baseline sample of 201 participants. Of these, 128 women completed the 6‐month follow‐up. Given the non‐random recruitment strategy and attrition at follow‐up, the possibility of selection bias cannot be fully excluded. Guided by the Fear‐Avoidance Model, the current study examines whether catastrophizing predicts depressive symptoms via pain interference. The variables and outcomes investigated are distinct from those used in previous research, thereby diverging from prior studies and addressing novel research questions.

Inclusion criteria required participants to be over the age of 18, to have received a diagnosis of endometriosis from a healthcare professional through surgery, gynaecological examination, pelvic ultrasounds, or magnetic resonance imaging, and to not be currently pregnant or menopausal. Additionally, participants needed to possess the ability to fluently understand Italian.

All participants took part voluntarily in the study and did not receive any compensation. Upon recruitment, patients were provided with information about the aims of the study and, after signing an informed consent form, were asked to complete an online survey questionnaire. In addition, participants were required to give an email address for further contact. After 6 months, participants were contacted again and asked to complete a second online questionnaire. In particular, at the baseline assessment (T1), participants were asked to provide sociodemographic and clinical information and to complete the Pain Catastrophizing Scale, the Italian version of the Multidimensional Pain Inventory, and the Patient Health Questionnaire (PHQ‐9), beyond reporting their levels of acyclic chronic pelvic pain. At the follow‐up assessment (T2), the PHQ‐9 was re‐administered, along with additional questions to verify whether any changes had occurred in the participants' conditions or current treatment regimen compared to 6 months earlier.

The study received approval from the Institutional Review Board of the Psychology Department at Sapienza University of Rome (Prot. N. 0000800).

### 2.2 Measures

#### 2.2.1 Sociodemographic Data and Clinical Information

At the moment of recruitment, women were asked to provide sociodemographic characteristics, including age, ethnicity, education, marital status, and occupation. In the survey, clinical information, including age at symptoms onset and diagnosis, how endometriosis was diagnosed, height and weight, menstrual characteristics (length of duration of menstrual cycle, presence of amenorrhea due to hormonal therapy), type of therapy, and characteristics of endometriosis (i.e., ovarian endometrioma and/or deep endometriosis), was asked to be reported.

#### 2.2.2 Pain Catastrophizing

Women completed the Italian version of the Pain Catastrophizing Scale (PCS—Sullivan et al. 1995; Italian version PCS‐I—Monticone et al. 2012), a self‐report measure that consists of 13 items assessing repetitive thinking, worry, and the inability to inhibit pain‐related thoughts (e.g., ‘I worry all the time about whether the pain will end’), the tendency to amplify the unpleasantness of painful situations and expectations of negative outcomes (e.g., ‘I become afraid that the pain will get worse’), and feelings of powerlessness and incapacity to cope with painful situations (e.g., ‘There's nothing I can do to reduce the intensity of the pain’). Participants were asked to indicate the frequency of thoughts and sensations described by each item when experiencing pain, answering on a 5‐point scale ranging from 0 (never) to 4 (always). Responses were averaged to produce an overall score, with higher scores reflecting greater pain catastrophizing. In our sample, Cronbach's alpha was 0.94.

#### 2.2.3 Pain Interference

Pain interference was measured using the Interference subscale of the Italian version of the Multidimensional Pain Inventory (MPI‐IV; Kerns et al. 1985; Italian version Ferrari et al. 2000). This subscale consists of 10 items evaluating the patient's perception of how pain interferes with various domains of daily functioning and the perception of behavioural limitations (e.g., ‘*In general, how much does pain interfere with your daily activities*?’). Responses to each item were rated on a 7‐point scale from 0 (no interference) to 6 (maximum interference), with higher scores indicating greater pain interference. In our sample, Cronbach's alpha was 0.92.

#### 2.2.4 Depressive Symptoms

To measure depressive symptoms, we used the Italian nine‐item version of the Patient Health Questionnaire (PHQ‐9; Spitzer et al. 1999; Italian version Mazzotti et al. 2003). This self‐report instrument allows for a rapid assessment of depressive symptoms severity in hospital and clinical settings. It consists of nine items assessing the presence of depressive symptoms over the past 2 weeks, corresponding to the criteria in the Diagnostic and Statistical Manual of Mental Disorders, fifth edition (DSM‐5), each rated on a 4‐point scale ranging from 0 (not at all) to 3 (nearly every day). Total scores range from 0 to 27, with scores between 5 and 9 indicating subthreshold depression, and a score of 10 is commonly used as the optimal cut‐off for clinically significant depressive symptoms (Mazzotti et al. 2003; Gilbody et al. 2007). Severity is further categorised as mild (5–9), moderate (10–14), or severe (15–27; MacArthur 2009). An example item included in the scale is: ‘*Over the past 2 weeks, how often have you been bothered by little interest or pleasure in doing things*?’ In our sample, Cronbach's alpha was 0.91. Although Mazzotti et al. (2003) expressed some caution regarding the use of the Italian version of the PHQ‐9—highlighting the need for careful cross‐cultural validation—the questionnaire remains widely used in both clinical and research settings due to its brevity and ease of administration. In our sample, internal consistency was excellent (Cronbach's alpha = 0.91), supporting its reliability in this context. In the present study, depressive symptoms at baseline were used as a covariate to control for pre‐existing depression, while depressive symptoms at follow‐up served as the main outcome variable.

#### 2.2.5 Numeric Pain Rating Scale

At the baseline assessment, participants were asked to report on pain symptoms, specifically acyclic pelvic pain. The severity of acyclic chronic pelvic pain was evaluated through a 0–10 numerical rating scale in which women were asked to mark the severity of their pain on a 10 mm numerical line referring to the average level of chronic pelvic pain where 0 = ‘no pain’ and 0 = ‘the worst imaginable pain’ experienced during the last month independently from period and intercourses. It is important to note that we focused on acyclic pelvic pain, as this type of pain is highly prevalent among individuals with endometriosis and tends to persist independently of the menstrual cycle, making it particularly relevant for assessing long‐term pain‐related outcomes (Vercellini et al. 2006).

### 2.3 Statistical Analysis

Data analyses were conducted using IBM SPSS Statistics version 26 (SPSS Inc., Armonk, NY, USA). The demographic, clinical, and psychological variables were described by their means (*M*) ± standard deviations (SD) or by the number of participants (*N*) with the percentage in parentheses.

The distribution of continuous variables was investigated using descriptive statistics. Univariate normality was examined via skewness and kurtosis; absolute skewness ≤ 2 and absolute kurtosis ≤ 7 were considered indicative of acceptable normality (West et al. 1995). Pearson correlations were conducted to examine the associations between study variables, and all results were reported as Pearson's *r* and *p*‐value.

An a priori power analysis using Monte Carlo simulations for indirect effects indicated that a minimum of 99 participants would be required to detect a medium effect size (0.30) with 95% power at *α* = 0.05. The achieved follow‐up sample of 128 participants exceeds this threshold. Nevertheless, this estimate is based on ideal assumptions and does not account for potential selection or attrition bias.

To examine whether pain interference mediated the relationship between catastrophizing and depressive symptoms, a mediation analysis was conducted using Hayes' PROCESS macro for SPSS (Model 4; A. F. Hayes 2017), which provided bootstrapping confidence intervals, model estimations, and conditional and direct effect computations. The method included 5000 bootstrap samples for coefficient and indirect estimation, and 95% percentile bootstrap confidence intervals (CI) were computed for the indirect effect, as recommended in recent methodological literature (Hayes and Scharkow 2013; A. F. Hayes 2018). The hypothesised model was built with Pain Catastrophizing (PCS‐I) as the independent variable, Pain Interference (MPI‐IV) as the mediator, and Depressive Symptomatology (PHQ‐9) as the dependent variable. Chronic Pelvic Pain (NRS), Age, Education, and Depression at baseline were entered as covariates. It should be noted that no direct statistical comparison between baseline and follow‐up depression scores was performed, as the focus of the analyses was on the prediction of follow‐up depressive symptoms, controlling for baseline levels.

We reported the standardised effect size (*β*) and the 95% percentile bootstrap CI for the indirect effect model. The mediation effect was considered significant when the 95% percentile bootstrap CI did not include zero (Preacher and Hayes 2008; A. F. Hayes 2018). Following Cohen's (1988) guidelines, standardised effect sizes (*β*) were interpreted as small (0.10), medium (0.30), and large (0.50). Notably, both the predictor (PCS‐I) and mediator (MPI‐IV) were measured concurrently at baseline (T1). Therefore, while the mediation model tests indirect effects consistent with a temporal process, the temporal ordering between these variables cannot be empirically established.

## 3 Results

### 3.1 Sample Characteristics

Of the initial sample of 201, 63.7% completed both baseline and follow‐up assessments conducted 6 months after the initial assessment. The analyses of the differences between those who participated only in the first assessment and those who completed both questionnaires revealed no significant differences: age (*t*_(199)_ = 0.023; *p* = 0.981), education (*t*_(199)_ = 0.010, *p* = 0.992), years between symptoms and diagnosis (*t*_(199)_ = 0.635; *p* = 0.526), chronic pelvic pain (*t*_(199)_ = 0.945; *p* = 0.345), catastrophizing (*t*_(199)_ = 0.312; *p* = 0.754), and depressive symptoms (*t*_(199)_ = 0.733; *p* = 0.464).

Tables 1 and 2 present the demographic and clinical characteristics of the sample, respectively. The average age of the sample was 36.0 (SD = 7.4) years, with an age range between 19 and 51. The totality of women were Caucasian (100%), with 98.7% of Italian nationality and 1.3% of another European country (i.e., Romania). Most participants had at least a high school education (89.1%) and were not in a stable relationship (60.2%). Overall, 81.3% of women were taking hormonal therapy, and 53.9% of them were in amenorrhea due to progestin or continuous oral contraception treatments. Regarding the presence of endometriosis, 35.2% of women had ovarian endometrioma, 43.0% had deep endometriosis (DIE), and 21.8% had both ovarian and DIE lesions. A large number of women (55.5%) had undergone a previous surgery. The mean diagnostic delay was 10.7 years (SD 8.1). Out of 128 patients, 68.8% exceeded the cut‐off for depression on the self‐report questionnaire.

**TABLE 1:** Sociodemographic characteristics of the patients (*n* = 128).

Variable	*M* ± SD or *N* (%)
Age	36.0 ± 7.4
Education	
High school diploma	51 (39.8%)
Bachelor's degree	27 (21.1%)
Master's degree	18 (14.1%)
Postgraduate courses	18 (14.1%)
Middle school certificate	13 (10.2%)
Elementary school certificate	1 (0.8%)
Marital status	
Married/committed relationship	51 (39.8%)
Single	41 (32.0%)
Divorced	36 (28.2%)
Employment status	
Employed	95 (74.2%)
Unemployed	20 (15.6%)
Student	13 (10.2%)
Ethnicity	
Caucasian	128 (100%)

**TABLE 2:** Clinical characteristics of the patients (*n* = 128).

Variable	*M* ± SD or *N* (%)
Menstrual cycle characteristics	
Amenorrhea due to hormonal treatment	69 (53.9%)
Between 21 and 30 days	39 (30.5%)
< 21 days of duration	8 (6.3%)
Between 31 and 35 days	8 (6.3%)
> 35 days of duration	4 (3.1%)
Obstetrics history	
Nulliparae	95 (74.2%)
Age at first childbirth	29.7 ± 5.1
Live birth (≥ 1)	30 (23.4%)
Miscarriage (≥ 1)	31 (24.2%)
Previous endometriosis surgical treatment	
Yes	71 (55.5%)
No	57 (44.5%)
Age at diagnosis	29.3 ± 7.1
Age at onset of pain symptoms	18.9 ± 8.9
Diagnostic delay	10.7 ± 8.1
Hormonal therapy	
Yes	104 (81.3%)
No	24 (18.8%)
Type of therapy	
Progestins	80 (76.9%)
Oestrogen‐progestins	24 (23.1%)
Type of endometriosis	
Ovarian endometrioma	45 (35.2%)
Deep endometriosis	55 (43.0%)
Both ovarian and deep	28 (21.8%)
BMI	23.7 ± 4.9
Chronic pelvic pain (NRS)	5.5 ± 2.9
Depressive symptoms	
PHQ‐9 at baseline	12.7 ± 5.7
PHQ‐9 ≥ 10 at baseline	88 (68.8%)
PHQ‐9 at follow up	12.9 ± 5.6
PHQ‐9 ≥ 10 at follow up	87 (68.0%)
Pain catastrophizing (PCS‐I)	29.0 ± 11.7
Pain interference (MPI‐IV)	3.98 ± 1.3

Abbreviations: BMI = body mass index, MPI‐IV = Multidimensional Pain Inventory, NRS = Numerical Rating Scale, PCS‐I = Italian version of the Pain Catastrophizing Scale, PHQ‐9 = Patient Health Questionnaire.

### 3.2 Matrix of Correlations

Table 3 reports the bivariate correlations among the variables of interest. The results showed that pain interference was positively correlated with catastrophizing (*r* = 0.59, *p* < 0.001), depression at baseline (*r* = 0.44, *p* < 0.001), and at follow‐up (*r* = 0.47, *p* < 0.001), and negatively correlated with education (*r* = −0.22, *p* = 0.03). Depressive symptomatology at follow‐up was positively correlated with catastrophizing (*r* = 0.49, *p* < 0.001), chronic pelvic pain (*r* = 0.37, *p* < 0.001), and negatively correlated with age (*r* = −0.19, *p* < 0.023) and education (*r* = 0.23, *p* = 0.028).

**TABLE 3:** Matrix of correlations (*n* = 128).

Variable	1.	2.	3.	4.	5.	6.	7.	8.	9.
1. Age	—								
2. Education	0.01	—							
3. Diagnostic delay	0.20*	0.16	—						
4. BMI	0.15	−0.05	0.09	—					
5. Chronic pelvic pain severity	−0.12	−0.19*	0.13	0.16	—				
6. Pain interference	−0.12	−0.22*	0.19*	0.13	0.42**	—			
7. Catastrophizing	−0.16	−0.20*	0.08	0.07	0.33**	0.62**	—		
8. PHQ‐9 at baseline	−0.20*	−0.22*	0.18	0.17	0.28**	0.44**	0.56**	—	
9. PHQ‐9 at follow up	−0.19*	−0.23*	0.11	0.17	0.37**	0.47**	0.50**	−0.69**	—

Abbreviations: BMI = body mass index, PHQ‐9 = Patient Health Questionnaire.

* *p* < 0.05.

** *p* < 0.001.

### 3.3 Mediation Model

**FIGURE 1:**
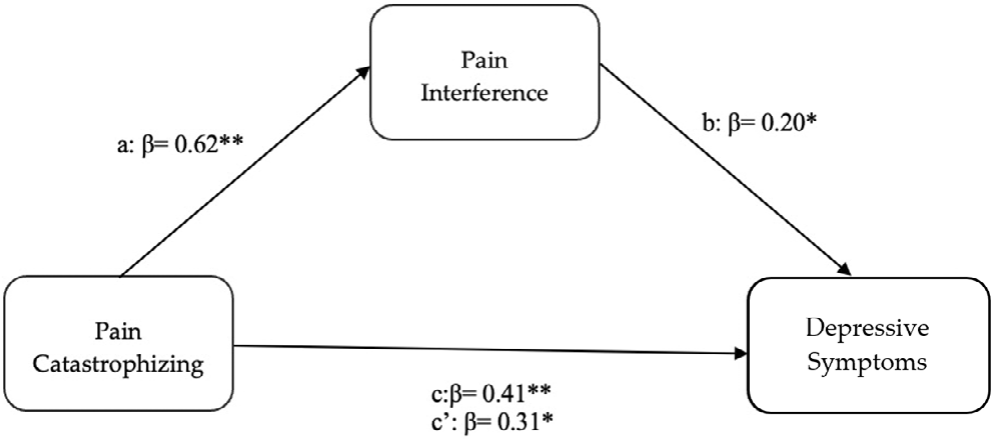
The mediational model (*n* = 128). *p < 0.05; **p < 0.001.

We examined the indirect effect of pain catastrophizing on depressive symptoms through pain interference, with chronic pelvic pain levels, age, and education as covariates. Preacher and Hayes (2008) bootstrapping estimate of indirect effects was employed. The overall model was significant (*F*_5,122_ = 21.262, *p* < 0.001, adj. *R* ^2^ = 0.325). Figure 1 displays the standardised regression coefficients among the model variables. The mediational model (Figure 1) revealed a significant indirect effect of the impact of pain catastrophizing on depressive symptoms through pain interference (*β* = 0.12, 95% bootstrap CI [0.003, 0.113]; unstandardized *a* = 0.07, *b* = 1.08, *ab* = 0.076). The total effect of pain catastrophizing on depression was significant (*β* = 0.41, 95% bootstrap CI [0.122, 0.270]; unstandardized *c* = 0.24). The direct effect of pain catastrophizing on depressive symptoms in the presence of the mediator was also significant (*β* = 0.31, 95% bootstrap CI [0.056, 0.236]; unstandardized *c*' = 0.162). Pain interference accounted for approximately 31% of the total effect.

## 4 Discussion

The present study aimed to explore the longitudinal relationship between pain catastrophizing, pain interference, and depressive symptoms in women with endometriosis. We hypothesised that high levels of catastrophizing would be associated with higher levels of pain interference, which in turn would predict higher depressive symptoms 6 months later. Findings indicated both a direct influence of pain catastrophizing on depressive symptomatology and an indirect relationship via pain interference. To our knowledge, these findings are the first to evaluate the effect of pain catastrophizing on depressive symptoms via pain interference in a longitudinal mediational model.

Previous longitudinal studies showed a significant association between this cognitive style and the severity of depressive symptoms in patients with other chronic pain conditions (Glette et al. 2021; Wood et al. 2023; Hawker et al. 2011; Trudel and Cormier 2024; Sullivan et al. 2001; Slepian et al. 2020). However, only a few cross‐sectional studies investigated this relationship in women with endometriosis (Zarbo et al. 2024; Evans et al. 2022; McPeak et al. 2018). Our study extends these findings, highlighting that pain catastrophizing has an impact on the psychological distress of this population, also over time, leading to a higher risk of developing depressive symptoms, even when controlling for depression and pain severity at baseline. Individuals who catastrophize tend to interpret pain experiences as more threatening and uncontrollable (Sullivan et al. 2001), which can contribute to negative emotions, a sense of helplessness, heightened emotional distress, and depressive symptomatology (Glette et al. 2021).

Our findings showed that the relationship between pain catastrophizing and depressive symptoms was partially mediated by pain interference with daily life. Specifically, findings confirmed that pain catastrophizing positively correlated with pain interference, indicating that individuals who exhibit elevated levels of pain‐related catastrophic thinking about their pain may also experience greater limitations in their daily activities (McPeak et al. 2018; Levang and Pukall 2024; Kalfas et al. 2022). This is in line with previous literature on non‐endometriosis patients, suggesting that catastrophizing can amplify the perceived severity of pain and contribute to maladaptive behaviours that may exacerbate functional impairment (Wilson et al. 2022; Schneider et al. 2022; Turner et al. 2016; Craner et al. 2016; Quartana et al. 2009). This finding is also consistent with the Fear‐Avoidance Model's emphasis on the role of cognitive factors, including catastrophic thinking, in amplifying the impact of pain on functional impairment (Vlaeyen and Linton 2000; Rogers and Farris 2022; Edwards et al. 2011). Catastrophic interpretations of pain sensations result in the development of pain‐related fear and the subsequent avoidance of activities perceived as potentially painful. Avoidant behaviours, in turn, may contribute to physical deconditioning, heightened pain sensitivity, and increased disability, perpetuating a cycle of pain and distress (Fritz et al. 2001; Sisco‐Taylor et al. 2022). However, it is important to recognise that, since in our study both catastrophizing and pain interference were assessed at the same time point, the directionality of this relationship cannot be definitively established. The results should, therefore, be understood within a correlational framework, limiting the ability to conclude the temporal or causal nature of the mediational pathway.

Moreover, the effect sizes differed in magnitude. The indirect effect of pain catastrophizing on depressive symptoms through pain interference was small, whereas the total and direct effects were of medium size. Pain interference accounted for approximately 31% of the total effect. Reporting both standardised (*β*) and unstandardized coefficients allowed us to contextualise these findings more accurately, as standardised values alone may obscure clinically meaningful changes in this population (Truax 1991). Even small indirect effects may nonetheless be clinically relevant since modest increases in pain interference can translate into significant deterioration in psychological adjustment among women with endometriosis (Fourquet et al. 2011; de Pessoa Farias Rodrigues et al. 2020).

Our findings also evidenced that perceiving more pain interference in daily functioning predicted depressive symptoms over time. This is consistent with previous studies evaluating the impact of chronic pain on the mental health of patients with endometriosis (Wang et al. 2021; Laganà et al. 2017). Indeed, when daily activities, social relationships, and occupational functioning are affected by pain, individuals may experience more frequent feelings of frustration, demoralisation, sadness, hopelessness, and loss of interest or pleasure in activities, all symptoms encompassed in depression (Dersh et al. 2002; Mills et al. 2019).

Finally, an important finding to be considered is the high prevalence of depressive symptoms among the sample, with over two‐thirds of participants exceeding the cut‐off for clinically significant depressive symptomatology. These findings align with the percentages reported in previous studies (Van Niekerk et al. 2022; van Barneveld et al. 2022; Gambadauro et al. 2019) and underscore the substantial psychological burden associated with endometriosis, highlighting the need for targeted intervention to address mental health concerns in this population. It is also noteworthy that both the depression levels and tendencies toward catastrophizing were negatively correlated with age. Young individuals' vulnerability may stem from the significant impact that the diagnosis of endometriosis may have on younger girls compared to older counterparts. Indeed, previous research proposed that younger patients experienced a greater impact of endometriosis on mental health (Ramin‐Wright et al. 2018; Simoens et al. 2012). This emphasises the need to address early tendencies toward pain catastrophizing, especially in younger cohorts. Cognitive‐behavioural therapy and Acceptance and Commitment Therapy are widely used intervention approaches that focus on identifying and challenging maladaptive thought patterns, including catastrophizing, to promote more adaptive coping responses and reduce emotional distress (Van Damme et al. 2004; McCracken and Vowles 2014; Trompetter et al. 2015).

The present study has several limitations. Firstly, depression was self‐reported, which may have introduced reporting bias. Individuals with higher levels of catastrophizing may be more prone to report increased frequency and severity of depressive symptoms. Ideally, incorporating clinically administered interviews or diagnostic assessments would enhance the accuracy and validity of depression assessment.

Secondly, catastrophizing and pain interference are measured concurrently at the same time point (i.e., baseline), limiting our ability to establish temporal precedence between these variables. It is important to acknowledge that these relationships may be bidirectional or cyclical. For example, pain interference may exacerbate catastrophizing through activity limitations, and depression may amplify catastrophizing, which in turn worsens mood and perceived well‐being. Future studies should explore this relationship longitudinally by incorporating a third time point between catastrophizing and depressive symptoms, allowing for a more comprehensive evaluation of these complex interactions.

Thirdly, the sample was assembled from different sources. Although the same inclusion and exclusion criteria were applied across all recruitment sources, the use of non‐random convenience sampling introduces the possibility of selection bias. Furthermore, while a power analysis using Monte Carlo simulations for indirect effects indicated that a minimum of 99 participants would be required—and our follow‐up sample of 128 participants exceeds this threshold—this estimate assumes ideal conditions and does not account for potential selection or attrition bias. The power to detect smaller or more complex effects may have been reduced, and the findings should be interpreted with appropriate caution.

Additionally, although the PCS‐I has been previously used in Italian studies involving women with endometriosis (e.g., Zarbo et al. 2024), it was originally validated in a population with chronic low back pain. Future studies should aim to establish its psychometric properties specifically within the endometriosis population.

Moreover, beyond catastrophizing, other variables not considered herein may further contribute to explaining depressive symptomatology. Factors such as pain acceptance, fear of movement and avoidance, and self‐efficacy for disease management could provide a more comprehensive and multifaceted understanding of how pain impacts women's mental health.

Finally, the generalizability of the findings is constrained by the sample's demographic composition, which was comprised entirely of women of Caucasian origin. Cultural differences in pain perception, coping strategies, and mental health stigma could influence the relationships examined in this study. Therefore, future research should enhance representativeness by including participants from minority communities and employing culturally and linguistically adapted measurement instruments. These efforts will be essential to improve the external validity of findings and better understand the complex interplay between pain, catastrophizing, and depressive symptoms across diverse populations.

## 5 Conclusions

The present study contributes to the growing body of literature on the psychological aspects of endometriosis. It sheds light on the relationship between pain catastrophizing, pain interference, and depressive symptoms in patients with endometriosis over time. In particular, findings indicated both a direct influence of pain catastrophizing on depressive symptomatology and an indirect relationship via pain interference, in line with the Fear‐Avoidance Model of pain. Future interventions tailored to alleviate pain interference and enhance the well‐being of women with this condition may take into account pain catastrophizing tendencies.

## Author Contributions

This study was designed by C.G., M.S. and M.G.P. The data were analysed by M.S. and C.D.G., and the results were critically examined by all authors. M.S. had a primary role in preparing the manuscript, which was edited by C.G. and C.D.G. All authors have approved the final version of the manuscript and agree to be accountable for all aspects of the work.
